# The Lived Body in Schizophrenia: Transition from Basic Self-Disorders to Full-Blown Psychosis

**DOI:** 10.3389/fpsyt.2015.00009

**Published:** 2015-02-03

**Authors:** Leonor Irarrázaval

**Affiliations:** ^1^Centro de Estudios de Fenomenología y Psiquiatría, Facultad de Medicina, Universidad Diego Portales, Santiago, Chile; ^2^Section Phenomenological Psychopathology and Psychotherapy, Psychiatric Department, University Clinic Heidelberg, Heidelberg, Germany

**Keywords:** schizophrenia, phenomenology, embodiment, self-disorder, intersubjectivity

## Abstract

This paper provides the results of a phenomenological study of patients with schizophrenia during their first psychiatric hospitalization. The study aims at clarify aspects related to the diagnosis of schizophrenia and to reach a greater understanding of the illness, with a view to contribute to prevention and psychotherapeutic intervention models. First, the paper offers a description of the patients’ “disembodiment” manifested in acute phases of schizophrenia. Second, it presents a description of the subjective anomalies that may be considered as disorders of “ipseity” or of pre-reflexive self-awareness. Third, the description is extended to encompass secondary disturbances to processes of establishing consensual intersubjectivity that lead to difficulties in shared communication practices and a progressive withdrawal from the intersubjective world. The conclusion states that a structural element, a key part of the personal processes involved in schizophrenia, is the diminishment of self-presence in experience, which manifests on both individual and social levels.

## Introduction

Traditionally, in the field of phenomenological psychopathology disorders of the self have been stressed as the essential clinical characteristics of schizophrenia. One hundred years ago, Jaspers was already describing them among the dimensions of self-awareness (1). Nevertheless, only in recent years has their importance been widely recognized, probably due to the increasingly significant focus on the early detection and prevention of psychosis (2). Neither there has been any great interest in this field in explaining nor understanding how disorders of the self are manifested on the social level. Again, it is only recently that hallucinations have begun to be considered as elements worthy of research in phenomenological approaches to intersubjectivity (3, 4).

Within the framework of contemporary psychopathological phenomenology, schizophrenia has been regarded as a paradigmatic disturbance of embodiment and intersubjectivity (5–12). It can be argued that its disorders are mainly manifested on two levels (13): (1) a disorder of “ipseity” or of pre-reflexive self-awareness that manifests as a diminishment of the first-person perspective tacitly given in experience, and (2) a secondary disturbance in the consensual processes of intersubjectivity, leading to difficulties in shared communication practices and in finding a place for oneself in the social world.

This paper provides the results of a phenomenological study of patients with schizophrenia during their first psychiatric hospitalization. First, it provides a description of patients’ “disembodiment” manifested in acute phases of schizophrenia. Second, it presents a description of the subjective anomalies most commonly experienced by these patients that may be considered as disorders of “ipseity” or of pre-reflexive self-awareness. Third, the description is extended to encompass secondary disturbances to processes of establishing consensual intersubjectivity that lead to difficulties in shared communication practices and a progressive withdrawal from the intersubjective world.

The study aims at clarify aspects related to the diagnosis of schizophrenia and to reach a greater understanding of the illness, with a view to contribute to prevention and psychotherapeutic intervention models. From this viewpoint, it seems appropriate to use methods that attempt to characterize not only the patients’ symptomatic disturbances manifested in acute phases of schizophrenia but also the anomalous self-experiences, which precede the onset of positive symptoms, thus broadening the scope of exploration to areas not taken into account in the criteriological manuals of diagnostic systems diagnostic statistical manual of mental disorders (DSM) and international classification of diseases (ICD) (14).

The manuscript is focused on the description of the subjective phenomena manifested among patients in different phases during their first psychiatric hospitalization, taking illustrative examples of three cases corresponding to a variety of schizophrenia subtypes. Cases 1, 2, and 3, as they appear in the paper, correspond to patients with diagnoses of disorganized-type, paranoid-type, and catatonic-type schizophrenia, respectively.

## Materials and Methods

### Study design

The study was developed within the qualitative paradigm, it being an explorative–descriptive type of study. This type of studies proceeds with inductive logic: in other words, both hypotheses and analysis categories are developed as the study progresses, and emerge from the data itself [Danhke, 1989 quoted in Ref. (15)].

The so-called “critical case sampling” criteria was used, where the interest in an in-depth approach to the phenomena means working with few cases, with representativeness not being of key importance for these purposes. An in-depth approach allows to access to the essential aspects of the personal experience, aiming at a greater understanding of the phenomena under research (16–18).

Data gathering were performed by means of semi-structured interviews, which are characterized by the use of eminently “open” research questions. Less structured methods allow for the emergence of ideographic descriptions, personal beliefs, and meanings, focusing on “how” the psychological processes occur (19).

The study proceeded with caution to avoid the bias commonly seen as a threat to the validity of qualitative data (20–22) (see Procedures and Analysis). All the interviews were recorded on video and fully transcribed for subsequent analysis. Extracts of the patients’ accounts were kept literally in quotes.

### Participants

The broad study covered a total of 15 patients with schizophrenia during their first psychiatric hospitalization. All of them were males, aged between 18 and 25. At the time of the interviews, the patients were receiving the usual pharmacological treatment for the diagnosis of schizophrenia.

Additional inclusion criteria were the following: (1) accessibility to the sample, (2) homogenous sample (23), and (3) earlier first onset and higher risk of developing schizophrenia in men (24).

Illustrative examples are provided of three cases, which were selected from the broader homogenous sample due to the variety of subtypes. Cases 1, 2, and 3, as they appear in the paper, correspond to patients with diagnoses of disorganized-type, paranoid-type, and catatonic-type schizophrenia, respectively.

### Instruments

#### In-depth interviews

In-depth interviews were used to gather qualitative data from the first encounter with the patients. These interviews had open questions aimed at allowing for a natural manifestation of the patients’ accounts. For the first encounter, the recommendations on interviews for the phenomenological diagnosis of schizophrenia were taken into account (25).

#### Examination of anomalous self-experience

The examination of anomalous self-experience [EASE; (26)] is a semi-structured interview for the phenomenological examination of disorders of the pre-reflexive self, postulated as early markers or basic phenotype of the schizophrenic spectrum (27). The EASE explores a variety of anomalous self-experiences, which typically precede the onset of positive symptoms and which also often underlie negative and disorganized symptoms (2).

#### Positive and negative syndrome scale

The positive and negative syndrome scale [PANSS; (28)] is a rating scale used for measuring symptom severity of patients with schizophrenia. The name refers to the two types of symptoms: positive symptoms, which refers to an excess or distortion of normal functions (e.g., hallucinations and delusions), and negative symptoms, which represents a diminution or loss of normal functions.

### Procedures and analyses

Five encounters with the patients were carried out. These encounters were coordinated throughout the three following phases.

#### Phase I

A first encounter to record the patients’ self-experiences manifested in acute phases of schizophrenia was carried out 1–2 weeks after hospitalization (30–45 min interview), following the confirmation of the diagnosis of schizophrenia in accordance with expert judgment and the standard diagnostic criteria of DSM-IV-R (29) and ICD-10 (30).

The patients’ accounts of the disturbances of self-experience and body alienations manifested in the acute episodes were summarized in corresponding descriptions containing the essential structure of the transcripts, which were obtained with the “Descriptive Phenomenological Method in Psychology” (31), by following five steps: (1) the researcher reads the entire transcript in order to gain an overall sense, (2) the same transcript is then read more slowly, and underlined every time a transition in meaning is perceived, providing a series of units constituting meaning, (3) the researcher then eliminates redundancies and clarifies the meaning of the units, connecting them together to obtain a sense of the whole, (4) the arising units are expressed essentially in the language of the subject, revealing the essence of the situation for him, and finally, (5) there is the summarizing and integrating of the achieved understanding in a description with the essential structure of the transcript.

#### Phase II

Two following encounters to carry out the “EASE” (26) took place after 1 month of hospitalization (30–45 min per each interview), when patients did not score with “positive” symptomatology on the “PANSS” (28).

The EASE manual was translated into Spanish by experts in phenomenological psychopathology, and advised by professional translators. The Spanish version was evaluated by the research team of lead author Parnas, who subsequently formally authorized its publication (32).

The interviewer must pass an EASE Introductory Course, which covers the following components: (1) a 1-day theoretical seminar, (2) a number of supervised interviews, and (3) a provisional assessment of reliability. This author attended the EASE Introductory Course on September 12th to 14th, 2011, at the Mental Health Center Hvidovre, Broendby, Denmark (see www.easenet.dk).

#### Phase III

Finally, two further encounters were held 1–2 months after hospitalization to perform the life story interviews (30–45 min per each interview). Transcripts were analyzed by peer researchers, both clinical psychologists with a specialty in psychotherapy. To avoid bias, each researcher previously made a separate analysis and then met for the co-analysis, ensuring with this procedure the validity of the qualitative research.

##### Note

More broadly speaking, the study includes a section on the patients’ life stories, which has been published in a complementary paper focused on analyzing the interpersonal processes involved in schizophrenia [see Ref. (33)]. Therefore, results of Phase III of the broad research have not been included in this paper.

### Ethical issues

The broad research, covering 15 patients with schizophrenia during their first psychiatric hospitalization, was regarded as entailing no physical, psychological, or social risks for the subjects involved, based on the Declaration of Helsinki principles, the Council for International Organizations of Medical Sciences (CIOMS) 1992 International Ethical Guidelines for Biomedical Research Involving Human Subjects, and the 1996 International Conference on Harmonization (ICH) Good Clinical Practice guidelines, by the following Ethics Committees: (1) Research into Human Beings Ethics Committee of the University of Chile’s Medical Faculty, dated January 19, 2011. (2) Ethics Committee Research of the Psychiatric Hospital, dated August 2, 2012. (3) Ethics Committee Research of the North Metropolitan Health Service (Santiago, Chile), dated August 16, 2012.

The Ethics Committees also approved the patients’ and their tutors’ (legal representatives) consent documents. In this regard, the following ethical aspects were taken into account: (1) consent was informed and obtained from the patients’ tutors by the attending doctor at Phase I of the study, considering that as a patient affected by an acute episode of schizophrenia, his competence or capacity is diminished and he must be authorized to participate. (2) Consent was obtained directly from the patients at Phase II of the study. (3) Pseudonyms were employed to protect the identity of the patients and ensure confidentiality (internal codes were used for each patient to replace their original names).

#### Note

Careful attention was paid in this paper to the protection of the patients’ anonymity. Identifying information such as dates, locations, and hospital numbers was avoided.

## Results

### Disembodiment

Phenomenology has developed a distinction between lived body (*Leib*) and physical body (*Koerper*), or body subject and body object. The former is the body experienced “from within,” my own immediate experience of my body tacitly given in the first-person perspective. The latter is the body thematically investigated “from without,” or from a third-person perspective, for example, by natural sciences such as anatomy and physiology (12, 34).

The term “embodiment” does not refer to biological or physical aspects of the body, such as the organs or the functionality of biological systems, but rather to a dimension that could be described as existential or experience-based: the embodied subject with regard to the intersubjective world.

In this regard, the embodiment-related dimension of mental illnesses does not manifest itself as impairment at an organic or biological level, but rather as a disturbance in the experience of one’s own body.

The disturbance of embodiment characteristic of schizophrenia has been called “disembodiment” (9, 10, 12). This does not literally imply a division, divorce, or separation between mind and body, but rather a “subjective distancing.” The body loses its tacit central role and does not serve as a medium of one’s involvement in the world any more (13).

The body loses its familiarity, resulting in various forms of body alienations. Single bodily sensations, movements, feelings, perceptions, or thoughts no longer flow naturally as mediating processes of embodiment but appear as obstacles to awareness with object-like qualities (10).

In acute phases of schizophrenia, the mediacy of the body is affected as a whole. And rather than being tacit and transparent, the body takes on layers of opacity. The body loses its transparent quality, its tacit experience-based function, turning into an object of observation, of thinking, of concern, and thus ends up being the “thematic” of an impediment or a problem.

#### Examples

##### Case 1

This first example is of a patient with disorganized-type schizophrenia who, although he considers himself to be a “normal” person, begins to recognize a “repetitive failure.” It is primarily the mediating process of thinking that has become the main impediment for the patient.

The trouble I was having was heart pain, together with headaches, nothing more. I thought that it would be another sort of hospitalisation [not psychiatric]. Now, I admit that my experiences were strange: the voices I hear inside my head. These voices are as if my own voice appeared inside my head saying a lot of terrible things, really bad things, like ‘evilisations’ (patient’s neologism). Also, other things are repeated in my head like an echo, for example, lately, ‘discharge me, discharge me soon’. Most of the voices I hear repeat things I don’t understand, I don’t know what they mean. Also, I get the feeling that there is like a sound repeating itself in my body, in my throat, going like m, m, m, m.

Patients with schizophrenia take an external position of self-observation, becoming a mere spectator of themselves. This tendency to take external or “disembodied” perspectives with regard to oneself is what eventually leads patients to create pathological attributions or explanations of their emotional processes or of their own bodies (8).

##### Case 2

The following example illustrates the “pathological” explanations configuring the delusion of a patient with paranoid-type schizophrenia. The patient has not been able to find a convincing explanation for the fear he feels, which has become his major impediment. Instead of attributing the fear to his existential processes or biographical circumstances, the patient assumes an external explanation: the biblical time of the “Tribulation.”

I wanted to find a way to overcome the fear, but now I don’t know what to think any more, what is causing this fear. I think I could be delivered over to the Tribulation –the Tribulation is a biblical time of pain, that I could be delivered over to pain. I’d like to find an answer in the Bible, what I’ve got to do, how to live and how to face up to the fear, and what’s going to happen. However, what I’ve read in the Bible hasn’t been enough: it doesn’t show me way, the exact way I should take for the situation I think that I am experiencing. I wish that the Bible could tell me what you do in the Tribulation, if I was in that time, that it told me in light of this fear to do this or that, to face up to it, don’t be afraid, I’ll be with you.

In acute phases of schizophrenia, patients’ accounts concentrate on (or are limited to) their anomalous self-experiences or body alienations. In other words, patients’ accounts lie outside the time-space dimension of the social context and exclude the personal history. Body alienation appears to be the way in which the desubjectivised accounts find concrete form (or are materialised).

##### Case 3

This example is of a patient with catatonic-type schizophrenia. The body alienations are explained as “demonic possessions,” which seems to increase the patient’s pathological condition.

Well, what happens is that I had strange things, possessions. I got like cramps back in my head, my body began to cramp up, and I began to shout, to cry and to scream like a devil. I feel that someone is possessing my body. I thought it could be a satanic influence. I don’t know: I did a bit of research and the spirit gets in when someone is too depressed, when you’re depressed is when the spirits get in. That makes me scared. It’s something that’s out there, something imminent, then it becomes pressing for me, when it begins to talk for me. I even hear a buzzing on my left side, inside my head.

For instance, the pervasive fear experienced by paranoid patients becomes the predominant external threat that acquires the characteristics of a delusion. These patients have a constant fear of being harmed or killed by others: somehow, it is the world and “the others” that have become unreliable or threatening, as shown in the following example.

##### Case 2

About three months ago I began to feel persecuted, persecuted by people. My house was the only place I felt safe, but for a few weeks now I have even begun to feel unsafe at home. The idea that they can hurt me comes from the fear I feel and I think that the worst thing would be for them to kill me somehow, like stabbing me, for example. Anything can happen these days.

Something curious about psychotic states is that not only do patients see the world through the framework of their delusions but also that this view is irrefutable to them. This implies a difficulty to enter into an open interaction that takes into account the viewpoint of the other person. In a way, the patient stands outside of the intersubjective dialectic provided by the second-person perspective (35), like in the next example.

##### Case 2

I found it suspicious that my workmates didn’t want to have their tea where I had mine, so I thought they had put something in it, some spit maybe, or that they had poured some vinegar in it. I saw a teapot and a thermos, and if I took it out of the thermos, they took it out of the pot, and that seemed odd to me so I threw away the tea. They didn’t like me, I thought, because of the way I am, not very friendly or ‘chatty’. I asked my workmates if they had put anything in my tea and they said no, nothing, but I didn’t believe them. The matter of food is a difficult one for me.

Besides, in the psychotic state, it could metaphorically be said that there is “no-border” between one’s self and others, as would normally be the case. There is a loss of the ability to distinguish between self-created meanings and those created by others, and to realize, as we normally do, that inanimate objects cannot actually create meanings or messages (e.g., self-referential messages patients discern from what they have heard on the radio, watched on television, or read in the newspapers). It could also be noted that there is a pervasive and unnoticed “self-referentiality” in experience in this state.

### Ipseity

Current psychopathological phenomenology has gained ground, emphasizing that the roots of mental illness are to be found in the patient’s pre-reflexive or pre-thematic experience (36). From this viewpoint, it is argued that schizophrenia involves a particular disturbance of basic self-awareness, more specifically a disorder of “ipseity,” normally occurring tacitly or pre-reflexively.

Ipseity (ipse is Latin for *self* or *itself*) refers to the fundamental configuration of self-awareness, corresponding to the first-person perspective tacitly given in experience (2, 37, 38). This perspective is oriented intentionally “from within” toward the world, and presupposes an immediate sense of “mineness” of the experience, as “being mine” or as “my own doing,” i.e., a quality of “personally belonging” or of “personalization” (1). The diminishment of this perspective mode of self-experience would lead to characteristic anomalies or basic disorders of the schizophrenic spectrum.

From the phenomenological examination of patients’ experiences (26, 32), disorders of ipseity are predominantly manifested in the domain of “cognition and stream of consciousness.” They are also frequently manifested in the domain of “bodily experiences,” like the sensation and/or perception of a “morphological change” of the body, as well as “mirror-related phenomena” (repeatedly looking at oneself in the mirror). Stereotypical conduct or motor interference, resulting from patients’ losing control of their own experience, is also manifested.

Within the domain of “cognition and stream of consciousness,” patients refer to “thought interference,” i.e., they refer to having experienced thoughts or imaginations that appear automatically, interrupting the main line of thought or interfering with it. Sometimes they intensify, ending up as “thought pressure”: a sense of many thoughts, lacking coherence with one another, appear in quick sequences without the patient being able to control them. Similarly, in some cases, patients have the feeling that their own thoughts are automatically (involuntarily) repeated or being in some way duplicated.

Thoughts are often experienced in a spatially localized way, and also with acoustic/auditory qualities. Frequently, there is a transition from experiencing thoughts on a quasi-perceptual level to external auditory hallucinations. In the beginning, patients hear their own thoughts not with their ears, but as their own voices inside their heads. With the diminishment of the sense of “mineness,” thoughts lose their familiarity, and patients now start to hear other voices inside their head (which no longer appear to them to be their thoughts). The voices are anonymous (impersonal): patients do not identify them with anyone in particular, and only fleetingly do they realize that these voices could be their own thoughts.

In full-blown psychosis, the experience is externalized, and the voices are heard as coming from the outside, thus acquiring the characteristics of a hallucination. The patient regards as an external reality something that is, in fact, part of his own fragmented experience. It could be said that, in the psychotic state, subjective experience is lived as “not as one’s own” and acquires an “inverted intentionality” (9, 39) as is the case of the next example.

#### Case 1

Sometimes I repeat my thoughts as if I were reading them, and I also hear voices on the radio, as if they were saying what I’m thinking: these are voices of unknown people who seem to be talking to me, but I don’t not know how or why they do so. Additionally, it sometimes seems to me that some television personalities repeatedly say things to me, all sorts of things, thousands of stupid things that I don’t understand, and nor do I understand why they’re doing this. Moreover, I heard a young man repeating all the things, I heard them everywhere, in the atmosphere, even with my mouth closed things were repeated any way.

Up to this point, the transition has moved from disorders of ipseity to disorders of “agency,” leading to an externalization of self-experience and loss of demarcation between the self and the environment (40). In this way, the subject gradually loses his self-presence in the experience. This transition occurs from momentary experiences of disembodiment to the more severe “depersonalization” (or desubjectivisation) manifested in the psychotic state. Here, the term “depersonalization” refers to severe self-disorders, i.e., thoughts, actions, or feelings occurring “with the awareness of their not being mine, of being alien, automatic, independent, arriving from elsewhere” [Ref. (1), p. 121].

### Intersubjectivity

Patients with schizophrenia display difficulties in naturally and spontaneously immersing themselves in everyday life, and find it particularly hard to grasp the “common sense” of situations (41–43). Generally, patients do not feel that they are fully participating or completely present in the world.

Misunderstandings and confusions about meanings are frequent in patients’ interactions with others. Synchronicity in social interaction seems interrupted mainly due to mismatches regarding “meaning coordination” processes (44) and difficulties in incorporating the perspective of others, as shown in the example below.

#### Case 2

It could be, like, someone says a word and, like, it appears bad to the other person: that’s what happens to me – words can have a double meaning. Just a while ago I spoke to someone and a slightly odd word came out. Like, he asked me why I didn’t have breakfast, and I told him it was because it was a bit of a pain. Then I thought that the person might be thinking that I was saying that he was a pain. I worry that the other person may take it the wrong way, because I don’t want to be a bother.

To a greater or lesser extent, patients display difficulties in making themselves understood or in explaining to others the experiences affecting them. In extreme cases, those patients that are especially self-absorbed or disconnected from the outside world (such as patients with catatonic-type schizophrenia) have great difficulties in articulating and expressing their experience, which remains to a large degree ineffable. This group of patients displays marked symptoms of affective flattening and autism.

Worth noting are the contradictory qualities of affection that are manifested among patients. Patients with schizophrenia are heterogeneous with regard to experience and expression of affection. Affective flattening appears less characteristic among paranoid patients.

In general, patients appear concerned with and anxious to understand what is happening to them, to recover and to quickly be discharged from their hospitalization. The majority refer to having anxiety crises, suicidal ideations, and suicide attempts. Nevertheless, the latter do not arise from a typically depressed state of mind, but rather from the desperation caused by patients’ experiences of self-alienation.

In the two examples below, patients regard their anomalies of self-experience and body alienations as an impediment or obstacle to live a normal life. Disorders of the self extend to include intersubjectivity, leading to a radical withdrawal from the social world, and to the extreme of contemplating suicide to put an end to their alienation.

#### Case 1

There are times when thousands of things are repeated, almost everything is repeated in my head, and then others when they disappear and things are normal, but then they come back again. Sometimes I couldn’t go out on the street, I had to stay in bed, just sleeping to get away from these thoughts. This has made it difficult for me to continue with my studies and concentrate. The situation is annoying and bad for me because I can’t live a normal life. It’s unbearable for me sometimes, and makes me think of killing myself, of hanging myself.

#### Case 3

I feel mental pressures, and it’s like they squeeze my brain, my entire brain. My thoughts are jumbled up, they’re a mess of ideas and thoughts. Reality gets distorted for me, too, I see it in a different way, like it was dream. This makes daily life difficult for me, living like this, living like this every day. I find it hard to relate to other people: in fact, I’ve really distanced myself from my friends. Because of my mental state, I don’t want to do anything: this bothers me, and makes me desperate, deep down. I am worried about my mental health state, not feeling normal. I’ve tried to kill my self several times. Before they brought me here I was going to jump off a mountain, I wanted to jump off a mountain because of the desperation.

## Discussion

### Key findings

A structural element, a key part of the personal processes involved in schizophrenia, is the diminishment of self-presence in experience. The subject gradually loses his self-presence in experience, which manifests not only on the individual level but also on the intersubjective level, leading to an early withdrawal from the social world.

On the individual level, the diminishment of self-presence in experience is a key element in the transition from basic self-disorders to full-blown psychosis. This transition occurs from momentary experiences of disembodiment to the more severe “depersonalization” (or desubjectivisation) manifested in the acute episode.

In early or prodromal phases, before “positive” symptoms occur, basic self-disorders are predominantly manifested in the domain of “cognition and stream of consciousness,” including “thought interference,” “thought pressure” and the feeling that thoughts are automatically being “repeated” or duplicated. Thoughts are often experienced in a spatially and localized way, and also with acoustic/auditory qualities.

Basic self-disorders are also frequently manifested in the domain of “bodily experiences,” like the sensation and/or perception of a “morphological change” of the body, as well as “mirror-related phenomena” (repeatedly looking at oneself in the mirror). Stereotypical conduct or motor interference, resulting from patients’ losing control of their own experience, is also manifested.

On the intersubjective level, major disturbances in the processes of synchronization with others are manifested. Patients do not feel at all participating or entirely present in the world. They show difficulties in dealing with consensual principles of understandability (common sense) and in incorporating the perspective of others (second-person perspective), which would appear to be important variables to consider in the symptomatological description of the schizophrenic spectrum disorders.

Patients with schizophrenia are heterogeneous with regard to experience and expression of affection. Affective flattening appears less characteristic among paranoid patients.

### Clinical implications

It is important to highlight the fact that hospitalization provides the first setting for the patients becoming conscious of their illness. This is the time when the illness is manifested, stemming from an initial psychotic break. This moment, which is critical in the prognosis, is the turning point, since, once discharged, patients return to the “non-place” that they occupied prior to hospitalization and, in a best-case scenario, they will occupy the place of the “sick person,” of the “schizophrenic.”

Medical-psychiatric intervention, which is the predominant form of intervention in the acute phase of schizophrenia, is mainly oriented toward reducing “positive” symptomatology. Nevertheless, looked at from a broader perspective, the symptomatological aspect of the acute phase is merely a sign or a signal of the seriousness of the overall situation affecting the patient. Thus, it can be seen that there is a need for diagnosis to involve more aspects of the patient’s life, in addition to those symptomatic aspects treated during hospitalization. This latter aim will require the effort of an ongoing interdisciplinary intervention.

Descriptions of disorders of the self would appear helpful in achieving a better understanding of the emergence of acute episodes of schizophrenia, by following the transition from basic disorders of ipseity to full-blown psychotic symptoms manifested in the patients’ experiences of “disembodiment.” Additionally, it would be also helpful to take into account the interpersonal scenario in which the psychotic episode emerges. This is, to contextualize the symptoms embedding them in the patients’ lives. What is more, to ensure a comprehensive understanding the configuration of schizophrenia, it would be necessary to examine disorders of the self in the light of patients’ life stories [see Ref. (33)].

Actually, in many cases, it is difficult to confirm a diagnosis of schizophrenia until well into hospitalization, or even until the illness is advanced. So, by broadening the scope of exploration of the patient’s experience to areas not taken into account in the DSM and ICD classifications, new descriptive elements are contributed that could potentially provide clarification for diagnosis.

### Future directions

Diagnostic statistical manual of mental disorders and ICD diagnostic classifications require review in light of approaches aiming for a greater understanding of the personal processes involved in schizophrenia (from the patients’ viewpoint) and that make it possible to explain aspects that remain unclear with regard to the trustworthiness of the diagnosis. These manuals spring from explanatory systems based on diagnostic categories according to a description of the apparent symptomatology, in which the assessment of subjective experience is basically excluded. However, patients’ accounts of their own anomalous experiences seem essential for diagnostic accuracy and therapeutic purposes (14).

Diagnosis, in the terms currently proposed, consists in a comprehensive approach to the overall functioning, both mental and bodily, of the person seeking help, encompassing not only symptomatic aspects but also his or her healthy potential, in addition to the life context. In this way, it seems appropriate to develop methods that attempt at developing a “person-centered” approach to diagnosis by employing a more comprehensive and holistic assessment of the patients’ condition (45).

Simply put, the subject of the study must be the person as a whole. This implies not only an in-depth review of the methodologies underlying traditional “objectivizing” diagnostic conceptualizations of mental illnesses but also a need to develop methodologies for the holistic study of patients, without losing sight of their corporality, complexity, and uniqueness (46).

From this viewpoint, the use qualitative methodologies for the study of schizophrenia and psychosis seem highly appropriate. These are just the clinical pictures that have not been sufficiently tackled from non-objectivizing approaches, and there is thus a significant lack of understanding of these phenomena beyond their apparent symptomatology.

Schizophrenia has been a central topic of research in phenomenological psychiatry, which aims at detailed descriptions of psychopathological experiences, and is committed to the in-depth analysis of symptomatic aspects. In consequence, phenomenological psychopathology research should also be able to illuminate key elements to incorporate in specific psychotherapeutic interventions for persons with schizophrenia.

## Conflict of Interest Statement

The author declares that the research was conducted in the absence of any commercial or financial relationships that could be construed as a potential conflict of interest.
